# Exploring end of life priorities in Saudi males: usefulness of Q-methodology

**DOI:** 10.1186/s12904-015-0064-5

**Published:** 2015-11-26

**Authors:** Muhammad M. Hammami, Eman Al Gaai, Safa Hammami, Sahar Attala

**Affiliations:** Clinical Studies and Empirical Ethics Department, King Faisal Specialist Hospital and Research Centre, P O Box # 3354 (MBC 03), Riyadh, 11211 Saudi Arabia; Alfaisal University College of Medicine, Riyadh, Saudi Arabia

**Keywords:** End of life priorities, End of life dis-priorities, Q-methodology, Score-averaging, Muslims, Saudi males

## Abstract

**Background:**

Quality end-of-life care depends on understanding patients’ end-of-life choices. Individuals and cultures may hold end-of-life priorities at different hierarchy. Forced ranking rather than independent rating, and by-person factor analysis rather than averaging may reveal otherwise masked typologies.

**Methods:**

We explored Saudi males’ forced-ranked, end-of-life priorities and dis-priorities. Respondents (*n* = 120) rank-ordered 47 opinion statements on end-of-life care following a 9-category symmetrical distribution. Statements’ scores were analyzed by averaging analysis and factor analysis (Q-methodology).

**Results:**

Respondents’ mean age was 32.1 years (range, 18–65); 52 % reported average religiosity, 88 and 83 % ≥ very good health and life-quality, respectively, and 100 % ≥ high school education. Averaging analysis revealed that the extreme five end-of-life priorities were to, be at peace with God, be able to say the statement of faith, maintain dignity, resolve conflicts, and have religious death rituals respected, respectively. The extreme five dis-priorities were to, die in the hospital, not receive intensive care if in coma, die at peak of life, be informed about impending death by family/friends rather than doctor, and keep medical status confidential from family/friends, respectively. Q-methodology classified 67 % of respondents into five highly transcendent opinion types. Type-I (rituals-averse, family-caring, monitoring-coping, life-quality-concerned) and Type-V (rituals-apt, family-centered, neutral-coping, life-quantity-concerned) reported the lowest and highest religiosity, respectively. Type-II (rituals-apt, family-dependent, monitoring-coping, life-quantity-concerned) and Type-III (rituals-silent, self/family-neutral, avoidance-coping, life-quality & quantity-concerned) reported the best and worst life-quality, respectively. Type-I respondents were the oldest with the lowest general health, in contrast to Type-IV (rituals-apt, self-centered, monitoring-coping, life-quality/quantity-neutral). Of the extreme 14 priorities/dis-priorities for the five types, 29, 14, 14, 50, and 36 %, respectively, were not among the extreme 20 priorities/dis-priorities identified by averaging analysis for the entire cohort.

**Conclusions:**

1) Transcendence was the extreme end-of-life priority, and dying in the hospital was the extreme dis-priority. 2) Quality of life was conceptualized differently with less emphasize on its physiological aspects. 3) Disclosure of terminal illness to family/close friends was preferred as long it is through the patient. 4) Q-methodology identified five types of constellations of end-of-life priorities and dis-priorities that may be related to respondents’ demographics and are partially masked by averaging analysis.

**Electronic supplementary material:**

The online version of this article (doi:10.1186/s12904-015-0064-5) contains supplementary material, which is available to authorized users.

## Background

Quality end-of-life care is increasingly viewed as a global public health problem as well as health systems problem in need of better information [1, 2]. This is related to the facts that end-of-life is commonly associated with substantial burdens on dying individuals and their families, [3, 4] that advances in medical science and technology continue to provide new management choices to healthcare workers, who may not be well acquainted with their patients’ priorities, and that there is ever growing belief in autonomy and personalized medicine. The Institute of Medicine Committee on Care at the End of Life suggested that a good death is one that is free from avoidable distress and suffering for patients and their families, in accord with the patients’ and families’ wishes, and reasonably consistent with clinical, cultural, and ethical standards [4].

Several North American and European studies explored end-of-life priorities from the point of view of patients with life-limiting illness, [5–21] outpatients with chronic diseases, [22–26] family care givers, [5–7, 14, 15] recently bereaved families, [8, 13, 15, 27–29] physicians, [8, 12, 13, 19, 30] non-physicians care providers, [8, 12, 13, 31] and the general public [19, 32–37]. Recently, similar studies conducted in non-Western countries showed fundamental differences, [36–38] probably related to the facts that priorities are contingent on facilitating circumstances and prior experiences, and that attitudes towards futility, uncertainty, suffering, life-prolonging, institutionalization, truth-telling, decision-making, and coping styles are culture-dependent.

Three instruments have been widely used to evaluate the dying experience and explore end-of-life priorities, namely, Quality of Dying and Death (QODD), [27, 39, 40] Preference About Dying and Death (PADD), [14, 15] and Positive diveRsities of European prIorities for reSearch and Measurement in end of life cAre (PRISMA) [32–37]. Most studies used independent rating, whereby respondents tend to attribute maximum importance to a large number of choices [14]. Further, most studies used traditional survey approaches and assessed importance of priorities by averaging across individuals, which often results in a homogenization that obscure individual differences in priorities’ hierarchy. Q-methodology, a special type of by-person exploratory factor analysis, is a process whereby respondents model their point of view by rank-ordering opinion statements into piles (Q-sort) along a continuum defined by certain instructions [41]. Using the Q-sorts as variables, it produces grouping of respondents who rank-ordered the statements into similar arrangements [42]. Q-methodology identified four types of perception of good death among South Korean university students [43]. Further, exploratory factor analysis revealed that four latent domains underlie the QODD questionnaire, symptom control, preparation, connectedness, and transcendence [40].

The aims of this study were to explore Saudi males’ opinions regarding end-of-life priorities and the usefulness of Q-methodology in this setting.

## Methods

This exploratory cross-sectional study was conducted in accordance with the ethical principles contained in the Declaration of Helsinki after approval of the Research Ethics Committee (REC) of the King Faisal Specialist Hospital and Research Center (KFSH&RC). All respondents provided verbal informed consent before participating in the study.

### Instrument development and validation

The study instrument (Q-set) was developed, validated, and piloted by the authors. The initial Q-concourse (collection of opinion statements to represent possible end-of-life priorities) was constructed in Arabic language based on published conceptual frameworks [12, 13] and instruments, including the 31 items of PADD questionnaire [14, 15], the 44 attributes of QODD questionnaire, [27, 39, 40] and PRISMA European survey questionnaire [32–37] as well as related Islamic literature. The collected statements were sorted into groups and collapsed into a Q-set that covered various thematic domains, with the aim of maximizing comprehensiveness, balance, and representativeness, and reducing redundancy. Three of the investigators independently evaluated the collected statements. When two or more statements conveyed the same or similar message as judged by the investigators, the redundant statements were removed. For example, the following pairs of statements from the QODD and PADD, respectively, were considered redundant: “Freedom from shortness of breath.” and “Breathing comfort.”, “Maintain a sense of humor.” and “Ability to laugh and smile.”, and “Freedom from pain.” and “Pain under control.” Disagreements among were resolved through discussion. The statements were randomly numbered, and each was printed on a separate card. Q-sorting requires respondents to arrange statements according to their subjective relative importance, using a systematic forced distribution. For the purpose of this study, this consisted of arranging the statements into graded priority, dis-priority, and non-priority groups. A sorting sheet was developed to record the chosen order of statements in a way that forces the Q-sort into the shape of a quasi-normal distribution. The sorting sheet had nine categories (1 extreme dis-priority, 5 non-priority, 9 extreme priority) with symmetrically distributed number of slots under each category: categories 1 and 9, 3 slots each, categories 2 and 8, 4 slots each, categories 3 and 7, 6 slots each, and categories 4, 5, and 6, 7 slots each (Additional file 1, Sorting Sheet & Instructions).

Criterion validity does not apply to Q-methodology because of its subjective nature. Content validity was examined thorough literature review and by eliciting domain experts’ advice. Reliability was studied through test-retest assessment, re-sorting after assuming certain extreme points of view (for example, placing absolute value on life quality or life quantity), and focused probing in an interview session following completion of Q-sorts. Participants were asked to Q-sort the same Q-set three times: first according to their point of view, then after assuming a certain extreme point of view, and finally according to their point of view for a second time (without prior knowledge that the Q-sorting will be repeated). The aim was to ensure that the results are reproducible and reflect the point of view of the sorter rather than being a function of the particular Q-set used. Intra-individual correlation coefficient was consistently more than 0.8 (*n* = 10). Qualitative evaluation of Q-sorts after assuming extreme point of view as well as focused probing indicated that the Q-sorts reflected sorters’ point of view.

The developed Q-set and sorting instructions were piloted on 20 Saudis of different demographics. The primary emphasis was to identify statements that are ambiguous, unclear, or leading, as well as to identify potential areas that were not covered by the initial Q-set. The Q-sorts used for validation or pilot testing were not included in this report. The final Q-set consisted of 47 statements in 8 thematic domains: symptoms and personal control (*n* = 7), treatment preferences (*n* = 5), whole-person-concerns (*n* = 8), moment of death (*n* = 5), family/friends (*n* = 6), achieving sense of completion/spirituality/religiosity (*n* = 5), preparation for death (*n* = 5), and relationship with healthcare professionals (*n* = 6). The first three domains are most related to life quality vs quantity concerns, the fourth and fifth to connectedness, the sixth to transcendence, the seventh to coping, and the eighth to information-disclosure and decision-making. An English translation (accuracy confirmed by back translation) of the final Q-set is available in Additional file 2, Q-Set Domains).

### Instrument administration

Each respondent was given the Q-sorting instructions, Q-set cards, a Q-sort grid, and a Q-sorting sheet. Respondents were requested to comment on their extreme choices immediately after completing their Q-sort. They were observed while Q-sorting, and time spent was recorded. Completeness of Q-sort (i.e., each statement is sorted only once) was checked and respondents were asked to correct any identified mistake.

### Sample size and sampling

Sample size was based on convenience and practicality, consistent with Q-methodology exploratory nature. KFSH&RC Saudi employees as well as patients and patients’ companions attending outpatient clinics were invited to participate through direct contact and advertisement. Saudi adults (≥18 year old) who had completed at least a high school education, who were able to understand the purpose and procedures of the study, and who provided informed consent, were eligible to participate. The study recruited both males and females, however, due to limitation of the statistical program used for Q-methodology analysis (maximum 120 Q-sorts), and the fact that analyzing mixed male and female Q-sorts obscured important gender differences; male and female Q-sorts were analyzed separately; only the results of males are reported here.

The following data were also collected: age, self-declared religiosity (compared to Muslims in Saudi Arabia; 5-point scale, much less to much more), general health (5-point scale, excellent to poor), life quality (4-point scale, excellent to fair), employment status (student, employed, self-employed, not employed), living arrangement (with spouse, with parents, with children, with other family members, alone), and death experience in family/close friends (last year, last 5 years, none in last 5 years). Respondents also rated six statements related to attitude toward death and one statement related to life satisfaction on a 5-point scale (1 strongly agree, 5 strongly disagree).

### Analysis

Data were verified by double entry and validity checks. As pre-specified in the study protocol, due to complexity of sorting the Q-set into nine categories, Q-sorts that took <20 min were not considered valid and were excluded from analysis. Q-sorts were analyzed by by-person centroid factor analysis (Q-methodology analysis), using PCQ for Windows (PCQ Software, Portland, OR, USA). Data analysis in Q-methodology involves sequential application of correlation, factor analysis, and computation of factor scores (Additional file 3, Factor Analysis). Centroids were extracted and then subjected to Varimax rotation to mathematically find a solution for which each Q-sort (respondent) has large loading, preferably on one factor (factor loading indicates the strength of respondent’s association with the identified factor or opinion type). In order to facilitate factor interpretation, some factors were, in addition, judgmentally rotated to minimize negative loading. Q-sorts with significant (*p* < 0.01) loading ≥ 0.38 on one, and only one, factor were considered definer Q-sorts for the factor. A model Q-sort for each factor was composed from statements’ scores calculated as weighted (based on factor loading) average across definer Q-sorts. This idealized Q-sort represented how a hypothesized respondent with 100 % loading on the factor would have ordered all the Q-set statements. Interpretation of factors involved comparing composite statement scores across factors and reviewing respondents’ post-sorting comments. Respondents who loaded significantly on one of the identified factors were compared as groups, in regards to their age, sorting time, religiosity, general health, life quality, employment status, living arrangement, death experience in family/close friends, attitude to death, and life satisfaction. Wilcoxon Signed Ranks, student t, Mann–Whitney, Kruskal-Wallis, and Fisher exact test were used to compare opinion types based on the variable examined (IBM SPSS Statistics 20). Two-tailed p values are reported.

## Results

Evaluable sorts were returned by 120 respondents (four respondents spent <20 min in Q-sorting and their data were excluded). Mean (SD) sorting time was 38.2 (16.6) minutes.

Main demographic data of respondents are summarized in Table 1. Mean age was 32.1 (9.8) year (range, 18 to 65); 52 % considered their religiosity about the same as other Muslims in Saudi Arabia; 88 % and 83 % self-rated their general health and life quality, respectively, as very good/excellent; 74 % were employed, 84 % were living with a spouse or parents, and 46 % had death experience in family/close friends in the previous 5 years. As shown in Table 2, 60 % of respondents strongly agreed/agreed that they often think about dying, 45 % that they don’t like to think about their own death, 38 % that they have intense fear of death, 42 % that they are afraid of having a long slow death, and 55 % that they are worried about uncertainty of what happens after death. Nevertheless, 99 % strongly agreed/agreed with the statement “I believe that heaven will be a much better place than this world.” Finally, 45 % strongly disagreed/disagreed with the statement “If I could live my life over, I would change almost nothing.”

**Table 1:** Demographics of Study Respondents (no. = 120)

Age-mean (SD), yr.	32.1(9.8)
Religiosity- no. (%)	
Much more	4 (3)
Somewhat more	12 (10)
About the same	62 (52)
Somewhat less	30 (25)
Much less	11 (9)
General health- no. (%)	
Excellent	50 (42)
Very good	55 (46)
Good	13 (11)
Fair	2 (2)
Poor	0 (0)
Life quality- no. (%)	
Excellent	39 (33)
Very good	60 (50)
Good	17 (14)
Fair	4 (3)
Employment-no. (%)	
Student	21 (18)
Employed	83 (69)
Self employed	6 (5)
Not employed	10 (8)
Living arrangement-no. (%)	
With spouse	56 (47)
With parents	44 (37)
With children	0 (0)
With other family members	5 (4)
Alone	14 (12)
Death experience in family/close friends-no. (%)	
Last year	31 (26)
Last 5 years	55 (46)
None in last 5 years	34 (28)

Religiosity (compared to Muslims in Saudi Arabia), general health, and life quality were self-declared. All respondents were Saudi nationals, males, Muslims, with more than high school education

**Table 2:** Attitude toward Death and Life Satisfaction (*n* = 120)

Statement	Strongly agree	Agree	Neither agree nor disagree	Disagree	Strongly disagree
I often think about dying.	23 (19)	49 (41)	31 (26)	8 (7)	9 (8)
I don’t like to think about my own death.	23 (19)	31 (26)	26 (22)	18 (15)	22 (18)
I have an intense fear of death.	17 (14)	29 (24)	37 (31)	17 (14)	20 (17)
I am afraid of having a long slow death.	27 (23)	23 (19)	44 (37)	11 (9)	15 (13)
The uncertainty of not knowing what happens after death worries me.	41 (34)	25 (21)	21 (18)	12 (10)	20 (17)
I believe that heaven will be a much better place than this world.	113 (94)	6 (5)	1 (1)	0 (0)	0 (0)
If I could live my life over, I would change almost nothing.	14 (12)	23 (19)	29 (24)	33 (28)	20 (17)

Data represent number (%) of responses for each category

### Averaging analysis

**Fig. 1:**
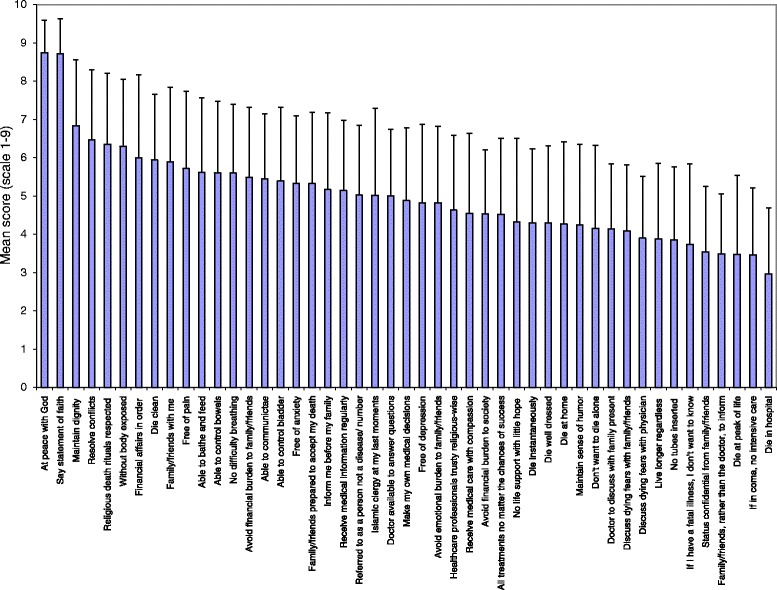
Respondents’ forced-ranking of 47 statements related to end-of-life care. Bars and error bars represent mean and SD of ranking scores on a scale of 1 (most disagreeable) to 9 (most agreeable). For full description of the statements, see text and Additional file 2

Respondents ranked 47 end-of-life opinion statements on a 9-point scale (1 extreme dis-priority, 9 extreme priority, 5 non-priority) following a systematic forced distribution (Additional file 1, Sorting Sheet & Instructions). Mean (SD) scores of the 47 statements are shown in Fig. 1. To facilitate interpretation of results, we considered that the cohort of 120 respondents, on average, held the ten statements with the highest mean ranking scores (8.7 to 5.7) as priorities, the ten statements with the lowest mean ranking scores (3.0 to 4.1) as dis-priorities, and the rest of the statements as non-priorities.

Four statements in the transcendence domain: “I want to die at peace with God.”, “I want to die being able to say the statement of faith.”, “I want to resolve any conflict before I die”, and “I want my religious death rituals to be respected.” received high priority scores (8.7 (0.9), 8.7 (0.9), 6.5 (1.8), and 6.4 (1.9), respectively). Three statements in the whole-person-concerns domain: “I want to die maintaining my dignity.” (6.8 (1.7)), “I want to die without having my body exposed.” (6.3 (1.8)), and “I want to die clean.” (5.9 (1.7)); and one statement in the symptoms and personal control domain: “I want to die free of pain.” (5.7 (2.0)) also received priority scores.

Four statements related to life quality/quantity, “If I go into coma, I do not want to be placed in an intensive care unit.”, “I want to die at the peak of my life.”, “I want to have no tubes inserted into my body.”, and “I want to live longer regardless of my medical condition.” received dis-priority scores (3.5 (1.8), 3.5 (2.1), 3.9 (1.9), and 3.9 (2.0), respectively). Two statements in the family/friends domain, “I want my family/friends, rather than my doctor to inform me about my impending death.” and “I want my medical status to be kept confidential from my family/friends.” also received dis-priority scores (3.5 (1.6) and 3.5 (1.7), respectively).

In the moment of death domain, one statement “I want to have my family/friends with me at my last moments.” received a priority score (5.9 (1.9)) and one statement “I want to die in hospital.” received the most extreme dis-priority score (3.0 (1.7)).

Finally, three statements in the preparation for death domain, “If I have a fatal illness, I don’t want to know.”, “I want to discuss my fears about dying with my physician.”, and “I want to discuss my fears about dying with my family/friends.” received dis-priority scores (3.7 (2.1), 3.9 (1.6), and 4.08 (1.7), respectively). However, statement, “I want to have my financial affairs in order before I die.” received a priority score (6.0 (2.2)).

### Factor analysis

Using the 120 Q-sorts as variables, we were able to extract 9 factors with eigenvalues higher than 1. However, it was not practical to apply all these factors for factor rotation or to meaningfully interpret them. By inspecting the scree plot (graphs eigenvalues against number of extracted factors) for areas of sudden decrease in eigenvalues and by logically analyzing the results after extracting 3 to 9 factors, we determined that the appropriate number of factors to extract was five. This five-factor solution accounted for 44 % of the total variance and 67 % of the 120 Q-sorts. Of the remaining Q-sorts, ten did not have significant loading on any of the factors, and 30 were confounded (loaded significantly on more than one factor).

There were three consensus statements and three differentiating statements. All the five factors (opinion types) gave the most extreme priority (rounded score 9) to “I want to die at peace with God.” and to “I want to die being able to say the statement of faith.” Respondents’ justification included “Without peace with God my life is worth nothing.”, “We are created to worship Him”, “If God is pleased, people will be pleased, so I will die with peace of mind.”, “It is the dream of every Muslim.”, “Because of my sins.”, and “To go to Paradise.” Some respondents cited the following verses from The Quran “I have only created Jinns and humans, that they may serve Me. No Sustenance do I require of them, nor do I require that they should feed Me. For Allah is He Who gives (all) Sustenance, − Lord of Power,- Steadfast (for ever).” (Verses 56–58, Chapter 51) and some cited Prophet Muhammad’s saying “He, whose last words are: there is no God but Allah will enter Paradise.” All opinion types gave the same dis-priority (rounded score 2) to “I want my medical status to be kept confidential from my family/friends.” Statement, “I want to have an Islamic clergy with me at my last moments.” differentiated opinion type I from the rest (rounded scores 1 vs. 5–7, respectively), statement, “I want to die being able to bathe and feed myself.” differentiated opinion type III from the rest (rounded scores 9 vs. 3–5, respectively), and statement, “I want to die being able to control my bladder.” differentiated opinion type V from the rest (rounded scores 1 vs. 5–7, respectively).

To facilitate interpretation of results, we considered that an issue was important to respondents belonging to a particular opinion type if its related statement received one of the seven highest (priority) or seven lowest (dis-priority) scores (corresponding to the two extreme columns in the sorting sheet, bilaterally) in the model Q-sort of the opinion type. Issues related to the rest of the statements were considered non-priority. As shown in Table 3, we were able to classify the five opinion types into rituals-apt, rituals-averse, or rituals-silent; family-caring, family-dependent, family-centered (both caring and dependent), self/family-neutral, or self-centered; monitoring-coping, avoidance-coping, or neutral-coping; and life-quality-concerned, life-quantity-concerned, life-quality/quantity-neutral, or life-quality & quantity-concerned. All of the five opinion types were highly transcendent.

**Table 3:** Opinion types identified by by-person factor analysis

Type I:	Type II:	Type III:	Type IV:	Type V:
Rituals-averse	Rituals-apt	Rituals-silent	Rituals-apt	Rituals-apt
Family-caring	Family-dependent	Self/family-neutral	Self-centered	Family-centered
Monitoring-coping	Monitoring-coping	Avoidance-coping	Monitoring-coping	Neutral-coping
Life-quality-concerned	Life-quantity-concerned	Life-quality & quantity-concerned	Life-quality/quantity-neutral	Life-quantity-concerned
Priorities in descending order				
At peace with God	At peace with God	At peace with God	At peace with God	At peace with God
Say statement of faith	Say statement faith	Say statement of faith	Say statement of faith	Say statement of faith
Maintain dignity	Financial affairs in order	Able to bathe and feed	Religious death rituals respected	Family/friends at my last moments
Resolve conflicts	Resolve conflicts	Maintain dignity	Die clean	Resolve conflicts
Avoid emotional burden to family/friends	Religious death rituals respected	Free of pain	Receive medical care with compassion	Free of pain
Avoid financial burden to family/friends	Family/friends with me	Able to control bowels	Inform me before my family	Religious death rituals respected
Without body exposed	Islamic clergy at my last moments	Without body exposed	No life support with little hope	Family/friends prepared to accept my death
Dis-priorities in ascending order				
Status confidential from family/friends	Free of pain	Family/friends, rather than doctor to inform	Family/friends, rather than doctor, to inform	Maintain sense of humor
Doctor to discuss with family present	If in coma, no intensive care	Status confidential from family/friends	Die in hospital	Die at the peak of life
Family/friends, rather than the doctor, to inform	Status confidential from family/friends	Discuss dying fears with physician	Avoid emotional burden to family/friends	Able to control bowels
Live longer regardless	No tubes inserted	Discuss dying fears with family/friends	Status confidential from family/friends	If in coma, no intensive care
If I have a fatal illness, I don’t want to know	Die in hospital	At the peak of life	Die well dressed	No tubes inserted
Healthcare professionals trusty religious-wise	If I have a fatal illness, don’t want to know	Die in hospital	Don’t want to die alone	Die well dressed
Islamic clergy at my last moments	No life support with little hope	If in coma, no intensive care	Die at home	Able to control bladder

### Opinion type I: rituals-averse transcendent, family-caring, monitoring-coping, life-quality-concerned

The eigenvalue and explained variance for opinion type I were 8.9 and 7 %, respectively. Thirteen respondents belonged to this opinion type only; 9 respondents belonged both to this and other opinion types.

In addition to the two common transcendence priorities, resolving conflicts before dying was a priority for opinion type I (in common with 2 out of the 4 other types). Respondents’ justifications included “So that I would forgive/be forgiven by those whom I aggrieved/who aggrieved me.”, “I don’t want any conflict after death.”, and “Good deeds/worships would not be accepted without forgiveness by others.” However, in variance with the other four types, having an Islamic clergy at time of death and receiving care from religiously trustworthy professionals were the most extreme dis-priorities. Justifications included, “What is the benefit?”, “It will make me feel depressed.”, “I think healthcare professionals have their own code of ethics regardless of their religions.”, and “There is no relation between religion and health issues.” Opinion type I could be described as family-caring, since it assigned priority scores to avoiding emotional and financial burdens to family/friends and dis-priority scores to “I want my family/friends rather than my doctor to inform me about my impending death.”, “I want my doctor to discuss any concerns related to my illness and care in the presence of my family/friends.”, and “I want my medical status to be kept confidential from family/friends”; suggesting that they would rather inform family/friends themselves in a way that would ease their emotional burden. Justifications included “They are believers, but I want to tell them in my own way.” This together with assigning a dis-priority score to “If I have a fatal illness I don’t want to know.” indicated a monitoring style of coping. Finally, opinion type I was life-quality-concerned, assigning priority scores to “I want to die maintaining dignity.” and “I want to die without having my body exposed.”, and dis-priority score to “I want to live longer regardless of my medical condition.” Justifications included, “God gave me dignity, I should maintain it.”, “If I die with dignity everything else is easy, everything follows dignity and respect.”, and “Long life with good deeds is good thing, but life just for staying alive is not important at all.”; suggesting that for this opinion type, transcendence is part of life quality.

It is of note that avoidance of emotional and financial burdens to family/friends, two priorities for this opinion type, were not among the ten priorities identified by averaging analysis for the entire cohort. Further, its two most extreme dis-priorities, having an Islamic clergy at the last moments and receiving care from religiously trustworthy professionals, were not among the ten dis-priorities for the entire cohort.

### Opinion type II: rituals-apt transcendent, family-dependent, monitoring-coping, life-quantity-concerned

The eigenvalue and explained variance for opinion type II were 12.4 and 10 %, respectively. Seventeen respondents belonged to this type only; 16 respondents belonged both to this and other opinion types.

This opinion type was also highly transcendent. However, in variance with opinion type I, it was described as rituals-apt as it gave priority scores to “I want my religious death rituals to be respected” and “I want to have an Islamic clergy with me at my last moments.” Justifications for the later included, “To remind me and presage me.”, “His presence will remind me of the statement of faith and to ask for forgiveness.”, and “Good thing to have, maybe he will help me (after God), to ease death and prepare to meet God.” Opinion type II could be described as family-dependent, as it gave priority score to “I want to have my family/friends with me at my last moments.” and dis-priority scores to “I want to die in the hospital.” and “I want my medical status to be kept confidential from my family/friends.” Justifications included, “I just want my family with me. It is not important where I die.” and “The opposite is true, they should know everything.” This opinion type strongly opposed “If I have a fatal illness I don’t want to know.” and strongly embraced “I want to have my financial affairs in order before I die.”; indicating a monitoring coping style. Justifications included, “I want to be prepared.”, “To the contrary, I should know.” “I need to know to seek treatment.”, “Illness is a test just as health is, knowing will rejuvenate faith.”, and “Fatal illness is a test and there would be big rewards for people who are patients.” for the first and “It affects my next life.”, “I will be questioned about it.”, “I don’t want conflict between my children.”, “It is a debt in my neck.”, “Financial matters may be a burden on me in my next life and in my grave.”, and “Debt is the most important thing one leaves after death, we will be stopped in front of God till the debt is given back.” for the second. Finally, it reacted strongly against “I don’t want to be kept on life support when there is little hope for a meaningful recovery”, “I want to have no tubes inserted into my body”, and “If I go into coma, I don’t want to be placed in an intensive care unit.” Justifications included, “I don’t care, I want cure at any price.”; “It is not a shame.”, “It is not important what is inserted in the body.”; “It is the doctor decision not mine.”, “It provides better care.”, and “When in coma, the decision is to the first of kin.” This opinion type even gave dis-priority score to “I want to die free of pain” (justified as “Pain would be expected when the soul departs from the flesh.”), indicating a strong life-quantity concern.

Having an Islamic clergy present at the last moments, a priority for this opinion type, and no life support with little hope, the most extreme dis-priority for this opinion type, were not among the 20 priorities and dis-priorities identified by averaging analysis for the entire cohort. Further, this opinion type gave dis-priority score to “I want to die free of pain”, which received a priority score by averaging analysis.

### Opinion type III: rituals-silent transcendent, self/family-neutral, avoidance-coping, life-quality & quantity-concerned

The eigenvalue and explained variance for opinion type III were 17.2 and 14 %, respectively. Thirty one respondents belonged to this type only; 24 respondents belonged both to this and other opinion types.

Despite being also highly transcendent, opinion type III was silent (gave non-priority scores) regarding “I want my religious death rituals to be respected.” and “I want to have an Islamic clergy with me at my last moments.” It could be described as self/family-neutral, as it was also silent regarding “I want to have my family/friends with me at my last moments.”, “I want to avoid being an emotional burden to my family/friends.”, and “I want to avoid being a financial burden to my family/friends.”; and gave weak dis-priority scores to “I want my family/friends, rather than my doctor to inform me about my impending death.” and “I want my medical status to be kept confidential from my family/friends.” It reacted strongly against “I want to discuss my fears about dying with my family/friends.” and “I want to discuss my fears about dying with my physician.” and was silent regarding “if I have a fatal illness, I don’t want to know.”; suggesting an avoidance coping style. In clear contrast to the other opinion types, the third highest priority for opinion type III was to be able to self bathe and feed. In addition, it gave priority scores to “I want to die maintaining my dignity.”, “I want to die free of pain.”, “I want to die being able to control my bowels.”, and “I want to die without having my body exposed.” and dis-priority score to “I want to die in hospital.”; suggesting a strong life-quality concern. Justifications included, “I don’t want to appear weak in the eyes of others.”, “I don’t want to be a burden on others.”, “Exposing the body is forbidden for Muslims.”, “I want to die at home.”, “Rarely people get out alive, more suffering in hospital.”, and “I want to die at home but near a hospital.” However, it also gave dis-priority scores to “If I go into coma, I do not want to be placed in an intensive care unit.” and “I want to die at the peak of my life.” and were silent regarding “I want to live longer regardless of my medical condition.”; suggesting an equally strong life-quantity concern as well. Justifications included, “It is the doctor who knows and should decide.”, “I want to use my life to worship God and obey my parents.”, “It is my goal to see my grandchildren and provide them with good life.”, and “I want to live longer.”

This opinion type contributed the largest number of respondents to the entire cohort. Nevertheless, its third and sixth highest priorities (being able to self bathe and feed and being able to control bowels) were not among the ten priorities identified by averaging analysis.

### Opinion type IV: rituals-apt transcendent, self-centered, monitoring-coping, life-quality/quantity-neutral

The eigenvalue and explained variance for opinion type IV were 8.6 and 7 %, respectively. Nine respondents belonged to this opinion type only; ten respondents belonged both to this and other opinion types.

In communality with the other four opinion types, this opinion type was highly transcendent. In agreement with opinion type II, it gave priority score to “I want my religious death rituals to be respected.” It also gave priority score to “I want to die clean.”, and thus was classified as rituals-apt. Justifications included, “Because cleanliness originates from/is part of belief.” Nevertheless, they were silent in regard to “I want to have an Islamic clergy with me at my last moments.” and gave dis-priority score to “I want to die well dressed.” Justifications included, “I wish I will die pure not well-dressed because I will go into soil.”, and “In the last moments, what is important is the inside (essence) not the outside (appearance).”; indicating different conceptualization of death rituals. Opinion type IV can be described as self-centered; it gave dis-priority scores to “I want to die at home.”, “I don’t want to die alone.”, and “I want to avoid being an emotional burden to my family/friends.”, and “I want to die in hospital.” and was silent in regards to “I want to resolve any conflict before I die.” and “I want to avoid being a financial burden to my family/friends.” Justifications included, “Place is not important, what is important is how to die.”, “Death in any place is the same death.”, and “Death is unavoidable; I wish I will die before my father, sibs, and friends.” Interestingly, in agreement with the other four opinion types, it gave dis-priority score to “I want my medical status to be kept confidential from my family/friends.”, probably here in order for the family/friends to be available to help. It gave priority score to “I want the doctor to inform me about my impending death before informing my family.” and dis-priority score to “I want my family/friends rather than my doctor to inform me about my impending death.”; suggesting a strong monitoring coping style. Finally, although it gave priority scores to “I want to receive medical care with compassion.” and to “I don’t want to be kept on life support when there is little hope for a meaningful recovery.” it was otherwise silent to issues related to life quantity and quality.

Three priorities (receiving medical care with compassion, being informed before family about impending death, and not to be kept on life support when there is little hope for meaningful recovery) and four dis-priorities (to die at home, not to die alone, to die well dressed, and to avoid emotional burden to family/friends) for this opinion type were not among the 20 priorities and dis-priorities identified by averaging analysis for the entire cohort.

### Opinion type V: rituals-apt transcendent, family-centered, neutral-coping, life-quantity-concerned

The eigenvalue and explained variance for type V were 6.9 and 6 %, respectively. Ten respondents belonged to this opinion type only; five respondents belonged both to this and other opinion types.

In agreement with opinion types II and IV, opinion type V was described as rituals-apt. Respondents justifications included, “Every human wants to be shrouded and buried according to his religion.” and “Because Islam respects the dead body.” Opinion type V was described as family-centered (both family-dependent and family-caring), as it embraced both “I want to have my family/friends with me at my last moments” and “I want to die knowing that my family /friends are prepared to accept my death”. Justifications included, “To pray for me and to forgive me.”, “To be comfortable.”, “I want to end with my family as I started with them.”, and “I expect to be happier with them.” It was silent in regards to coping style and information disclosure. However, it reacted strongly against “I want to die being able to control my bladder”, “I want to die well dressed.”, “I want to have no tubes inserted into my body.”, “If I go into coma, I don’t want to be placed in an intensive care unit.”, “I want to die being able to control my bowels.”, “I want to die at the peak of my life.”, and “I want to die maintaining my sense of humor.”; indicating a strong life-quantity-concern. Justifications included, “Controlling bladder does not mean anything to me.”, “When on death bed appearance is not important.”, “I want tubes inserted in my body to continue my treatment.”, and “Controlling bowels is not important at time of death.” Nevertheless, it gave priority score to “I want to die free of pain.”

One priority (having family/friends prepared to accept death) and four dis-priorities (to be able to control bladder, to die well dressed, to be able to control bowels, and to maintain sense of humor) for this opinion type were not among the 20 priorities and dis-priorities identified by averaging analysis for the entire cohort.

### Comparing normalized factor scores

Contrasting normalized scores for each of the 47 statement among the five opinion types supported our interpretation of the factors. For example, normalized score differences between opinion type I (life-quality-concerned) and opinion type II (life-quantity-concerned) in relation to “I do not want to be kept on life support when there is little hope for a meaningful recovery.”, “I want to die free of pain.”, “I want to die maintaining my dignity.”, and “I want to die free of depression.” were 1.79, 1.61, 1.07, 1.01, respectively. Similarly, score differences between opinion type I and opinion type V (life-quantity-concerned) in relation to “I want to die maintaining my dignity.” and “I want to live longer regardless of my medical condition.” were 1.52 and −1.57, respectively; and between opinion type II and type III (life quality and quantity-concerned) in relation to “I want to die free of pain.”, “I want to die having no difficulty breathing.”, “I want to die free of anxiety.”,, and “I want to die being able to bathe and feed myself.” were −1.84, −1.18, −1.15, and −1.08, respectively.

In the same vein, differences in normalized scores between opinion type I (rituals-averse) and opinion types II, IV, and V (rituals-apt) in relation to “I want an Islamic clergy with me at my last moments.”, and “I want to receive care from healthcare professionals whom I religiously trust” were −2.76 and −1.79, −2.01 and −1.75, and −1.91 and −1.90, respectively. Similarly, differences in normalized scores between opinion type III (avoidance-coping) and opinion types I, II, and IV (monitoring-coping) in relation to “I want to discuss my fears about dying with my family/friends” were −0.44, −1.03, and −1.04, respectively.

Although all five opinion types were highly transcendent there were quantitative differences. Opinion types I, II, and III were more transcendent than opinion types IV and V (normalized factor scores for “I want to die at peace with God.” 2.67, 1.90, 1.86, 0.63, 0.60, respectively, and for “I want to die being able to say the statement of faith.” 2.37, 1.82, 1.80, 0.63, 0.60, respectively).

### Association between identified opinion types and respondents characteristics

Table 4 summarizes the characteristics of respondents classified according to their opinion type. Compared to the other opinion types, Opinion type I had the highest mean age, the longest sorting time, and the lowest self-rated religiosity, general health, life quality, and life satisfaction. Opinion type II had the shortest sorting time and the best reported life quality, and was most agreeable to “I often think about dying.” Opinion type III had the lowest reported life quality. Opinion type IV had the lowest mean age, the best general health, and the highest life satisfaction, and was least agreeable to “I often think about dying.” Opinion type V had the highest self-rated religiosity.

**Table 4:** Characteristics of respondents per opinion type

	Type I (*n* = 13)	Type II (*n* = 17)	Type III (*n* = 31)	Type IV (*n* = 9)	Type V (*n* = 10)
Age (year)	36.4 (13.0)	36.1 (8.1)	31.4 (7.9)	26.3 (5.6)	29.4 (8.9)
Sorting time (minute)	41.8 (19.3)	31.0 (11.1)	39.8 (18.3)	38.9 (15.8)	35.0 (14.1)
Religiosity (1 least, 5 most)	2.9 (0.5)	3.4 (0.9)	3.1 (1.1)	3.4 (0.8)	3.7 (0.8)
General health (1 excellent, 5 poor)	1.9 (0.5)	1.7 (0.8)	1.7 (0.7)	1.3 (0.5)	1.7 (1.0)
Life quality (1 excellent, 4 fair)	1.9 (0.8)	1.5 (0.5)	1.9 (0.7)	1.7 (1.1)	1.7 (1.0)
“I often think about dying” (1 strongly agree, 5 strongly disagree)	2.7 (0.8)	1.8 (0.7)	2.6 (1.3)	2.9 (1.6)	2.7 (1.1)
“If I could live my life over, I would change almost nothing” (1 strongly agree, 5 strongly disagree)	3.6 (0.9)	2.9 (1.2)	3.1 (1.4)	2.3 (0.5)	3.0 (1.6)

Data represent mean (SD). Religiosity, general health, and life quality as self-declared. All respondents were Saudi nationals, males, and Muslims, and high school graduates

There were no significant differences among the five opinion types in relation to employment status, living arrangement, or death experience in family/close friends (Fisher’s exact test, *p* > 0.11). There was no significant difference in sorting time (ANOVA *p* = 0.42) and borderline significant difference in age (ANOVA, *p* = 0.057), which was due to a significant difference between opinion types I and IV (*p* = 0.04). Using Kruskal-Wallis test, there were no significant differences (*p* > 0.27) among the five opinion types in relation to religiosity, general health, life quality, life satisfaction, or death attitude, except in regard to statement “I often think about dying.” (*p* = 0.08). For this statement, there were significant differences (Mann–Whitney *U* test between opinion types II and I (*p* = 0.007), II and III (*p* = 0.04), and II and V (*p =* 0.02), with opinion type II having stronger agreement with the statement than the other 3 types. Further, there were significant differences between opinion types I and IV in life satisfaction and general health (Mann–Whitney *U* test, *p* = 0.006 and *p* = 0.03, respectively).

### Indifferent (non-priority) statements

The following 11 statements received non-priority scores both on averaging analysis and on factor analysis: “I want to die having no difficulty breathing.”, “I want to die free of anxiety.”, “I want to die free of depression.”, “I want to receive all available treatments no matter what the chances of success are.”, “I want to die being able to communicate with others.”, “I want to be referred to as a person not as a disease or a number.”, “I want to avoid being a financial burden to my society.”, “I want to die instantaneously.”, “I want to make my own medical decisions.”, “I want to have my doctor available to answer my questions.”, and “I want to receive medical information regularly from the medical staff.”

## Discussion

The aims of this study were to explore Saudi males’ opinions regarding end-of-life priorities and the usefulness of Q-methodology in this setting. We found that: 1) by averaging analysis, the extreme ten priorities were to, be at peace with God, be able to say the statement of faith, maintain dignity, resolve conflicts, have religious death rituals respected, not have the body exposed, have financial affairs in order, die clean, have family/ friends at last moments, and die free of pain, respectively. The extreme ten dis-priorities were to, die in the hospital, not receive intensive care if in coma, die at peak of life, be informed about impending death by family/friends rather than the doctor, keep medical status confidential from family/friends, not know if one has a fatal illness, not have tubes inserted in one’s body, live longer regardless of medical condition, discuss fears about dying with the physician, and discuss fear about dying with family/friends, respectively. 2) Q-methodology analysis classified 67 % of the respondents into five opinion types: rituals-averse transcendent, family-caring, monitoring-coping, life-quality-concerned; rituals-apt transcendent, family-dependent, monitoring-coping, life-quantity-concerned; rituals-silent transcendent, self/family-neutral, avoidance-coping, life-quality & quantity-concerned; rituals-apt transcendent, self-centered, monitoring-coping, life-quality/quantity-neutral; and rituals-apt transcendent, family-centered, neutral-coping, life-quantity-concerned. 3) Out of the extreme 14 priorities/dis-priorities for the five types, 29, 14, 14, 50, and 36 %, respectively, were not among the extreme 20 priorities/dis-priorities identified by averaging analysis for the entire cohort. 4) Eleven issues were identified as non-priority both on averaging analysis and Q-methodology analysis: to die having no breathing difficulty, anxiety, or depression, to receive all available treatments no matter what the chances of success are, to be able to communicate with others and referred to as a person, to avoid being a financial burden to society, to die instantaneously, to make own medical decisions, to have doctors available to answer questions, and to receive medical information regularly.

### Transcendence

Religious traditions often provide a framework for understanding death and dying as well as norms for end-of-life care. Islamic traditions, in common with Judeo-Christian traditions, view life as sacred and a mean to prepare the soul, and death as inevitable and a transition to another life. Our respondents were highly transcendent, eight of the top ten end-of-life priorities were clearly (to be at peace with God, be able to say the statement of faith, resolve conflicts, and have religious death rituals respected) or arguably (maintain dignity, not have the body exposed, have financial affairs in order, and die clean) in the transcendence domain. In contrast, in a North American study, coming to peace with God was ranked the second or third (after freedom from pain and presence of family), depending on the subgroup studied, [8] and in a European study, only 50 % of patients with advanced cancer declared a belief in any life after death [16].

According to Islamic traditions, when a person is dying, close relatives/friends kindly remind him/her of the vast Mercy of Allah and to say the statement of faith (there is no God but Allah). Dying rituals include having the dying person to lie or sit facing toward Mecca. After death, the body is ritually washed (modesty preserved), anointed, shrouded (men deal with male bodies and women with female bodies), prayed over, and buried with a simple rite. In this study, the importance of rituals to respondents was variable. Although three opinion types were rituals-apt, one was rituals-silent and one was rituals-averse, suggesting that to some, resolving issues of faith within oneself may be more important than social or interpersonal expressions of spirituality. Further, having religious death rituals respected, but not having an Islamic clergy at the last moments, was one of the overall priorities. Furthermore, respondents who belonged to the rituals-averse and rituals-silent opinion types had lower self-declared religiosity, which is likely related to ritual practices rather than strength of beliefs or inner piety, as these two opinion types had higher normalized factor scores for “I want to die at peace with God.” and “I want to die being able to say the statement of faith.” than two of the three rituals-apt opinion types. Interestingly, in a North American study, having funeral arrangements planned received stronger importance rating from patients compared to physicians, [8, 13] and meeting with a clergy received stronger importance rating from bereaved family members than patients or physicians [8].

### Life-quality vs. Life-quantity

Arguably, sanctity of life is related not only to its intrinsic value but also to some of its qualities, such as self-consciousness, ability to establish relationship, and ability to derive pleasure [30]. At the end-of-life, life-quality and life-quantity often become a dichotomy, and individuals and cultures often choose different points of equilibrium. Attitude of European neonatologists toward sanctity of life vs. quality of life varied both within and across countries in relation to religious background and religiousness [30]. More than 60 % of African [36, 37] and a higher percentage of European [35] general public preferred life-quality over life-quantity.

In our study, three out of the extreme ten dis-priorities indicated preference for life-quantity (not to receive intensive care if in coma, to die at peak of life, not to have tubes inserted in one’s body); and one dis-priority (to live longer regardless of medical condition) and four out of the extreme ten priorities (maintain dignity, die without having body exposed, die clean, and die free of pain) indicated preference for life-quality. Our results may be a reflection of the Islamic views of life sanctity, life as a mean to develop the soul, mandating relief of suffering whenever possible, and enduring and accepting irrelievable suffering as a spiritual reward. Nevertheless, Q-methodology showed that our respondents were not homogeneous in this regard. We were able to stratify respondents into life-quality-concerned, life-quantity-concerned (2 opinion types), life-quality and quantity-concerned, and life-quality/quantity-neutral. Preference for life quality was qualitatively associated with lower self-declared life quality, life satisfaction, and religiosity. A North American study on patients with advanced heart failure identified two distinct groups independent of functional or symptoms status, one preferring treatments that prolonged survival time and another that favored strategies that improved quality of life but reduced survival time [17]. However, a belief in divine intervention [9] and a lower spiritual well-being [24] have been associated with favoring life-sustaining treatment. Finally, wanting all available treatment may not necessarily reflect a preference for life-sustaining treatment, but rather distrust in medical culture or lesser familiarity with such treatments [8].

Conceptualizing what constitute life quality may differ among individuals, individuals in different roles, and cultures. Interestingly, to die free of pain was the tenth priority for our respondents, while other physiological (shortness of breath, ability to communicate) and psychological (anxiety, depression, maintaining sense of humor, be referred to as a person) indicators of life-quality were non-priorities. Further, to die free of pain was a priority for only two of the five opinion types, and in fact a weak dis-priority for one. On the other hand, to be able to self bathe and feed, to be able to control bowels, to die clean, and to die without having body exposed were variably among the top seven priorities of the five opinion types. The importance of having pain free death has been documented in previous studies. Freedom from pain was ranked as the most important by seriously ill patients, recently bereaved family, physicians, and non-physician care providers, [8] higher overall scoring of quality of dying and death by family members of ICU decedents was associated with control of pain, [28] being in pain was the most concerning of nine common end-of-life symptoms and problems to the general public, [37] and 63 % advanced cancer patients preferred to die in a state of unconsciousness induced by drugs [16]. However, in other studies keeping positive attitude was prioritized above having pain/discomfort relieved, [36] 8 % of patients and 6 % of healthy controls rejected an effective dose of painkillers if these might dull consciousness, [19] and being mentally aware received stronger importance rating from patients compared to physicians [8].

### Connectedness and preferred place of death

One of the extreme ten priorities (to have family/close friends at last minutes) and two of the extreme ten dis-priorities (to keep medical status confidential from family/friends, to discuss fears about dying with family/friends) indicated the importance of connectedness to our respondents overall. Q-methodology analysis was able to group respondents into, family-caring, family-dependent, family-centered (both caring and dependent), self-centered, and self/family-neutral. A study on terminally ill men revealed heterogeneity in views about presence of others at the very end of life [10]. Wanting family present at the time of death may reflect family dependence rather than family caring. In fact, family caring may result in hiding grave information and avoidance of family members’ presence at the time of death in order to minimize their emotional burden.

Overall, to die in the hospital was the most extreme dis-priority for our respondents, whereas to die at home was a non-priority. However, there was clear heterogeneity, to die in the hospital was a dis-priority for three opinion types, to die at home was a dis-priority for one opinion type, and two opinion types were silent as to the preferred place of death. Rather constant with our results, 51 % (Portugal) to 84 % (Netherland) of European general public, [32] 51 % of Kenyan general public, [36] and 67 % of Italian patients with advanced cancer preferred home as a place of death [16]. On the other hand, only 32 % of Namibian general public preferred to die at home while 48 % preferred to die in the hospital [37]. The preferred place of death appears to be, to some extent, role-dependent. For example, non-physician care providers were more likely than patients to agree with the importance of dying at home [8]. Preferring not to die at home may be related to lower medical coverage and symptoms control, a desire not to burden family resources, refusal to admit that a cure is not possible, and difficulty in taking care of the dead body.

### Information disclosure and coping

Five of the extreme 10 dis-priorities were related to information disclosure and coping style (not to know if one has a fatal illness, to be informed about impending death by family/friends rather than the doctor, to keep medical status confidential from family/friends, to discuss dying fears with family/friends, and to discuss dying fears with physician), indicating that overall respondents wanted to be informed about their fatal illness (but not to discuss it), to inform their family/friends about their medical condition, and to be informed before their family/friends. Nevertheless, the following were non-priorities, being able to communicate with others, having the doctor available to answer questions, and receiving medical information regularly. Our results are consistent with results in other cultures. In a North American study, preparation for the end of life was consistently rated as important by seriously ill patients, recently bereaved family, physicians, and non-physician care providers, [8] and about 56 % Kenyan and Namibian general public wanted to be told if they had limited time left without having to ask [36, 37].

Denying one’s death fits with a medical culture that defines death as a negative outcome in the fight against disease (rather than a transition to another life); therefore, one would fear that disclosing information about a terminal illness would remove hope and may not be favored over hiding it [13]. On the other hand, strong religious beliefs may not necessarily result in greater acceptance of death; spiritual coping can be through seeking control through a partnership with a higher power, giving up control to a higher power, or seeking spiritual support through the love and care of a higher power [9]. Arab countries, including Saudi Arabia have been characterized by high-context communication culture [44] and blunting coping style, [45] and it was hypothesized that in such cultures using “Western” reasonable patient standard of information disclosure may not be appropriate [46]. The results of this study and our previous study on desired information disclosure in clinical informed consent [47] are not consistent with such hypothesis. Interestingly, governing codes on disclosure of terminal illness to patients and families in Islamic countries vary considerably [48]. Adequate information disclosure provides an opportunity to complete unfinished business and say goodbye to important people. However, discussing death fears (with family, friends, or physician) may only remind the dying person of the unwelcomed event without added benefit to a previous disclosure. Seriously ill patients were less likely than recently bereaved family, physicians, and non-physician care providers to rate discussing personal fears as important [8].

### Decision making

Although not to know if one has a fatal illness was a dis-priority, making own medical decisions was a non-priority for our respondents. People value the right to not have their physical, emotional, and dispositional privacy invaded (negative liberty); however, the value of decisional privacy (positive liberty) is less certain [49]. Further, avoidance of shaping patients decisions (framing) by clinicians may be impossible, and it has been advocated that clinicians should aim to avoid restricting choice rather influencing choice [49, 50]. In contrast to the results of the current study, we found that Mill’s individual autonomy was the patients’ preferred model for clinical informed consent, [51] which is consistent with the observations that the more severe the illness, the less patients wished to make decisions themselves [52] and that having as much information as you want was prioritized above choosing who makes decisions about end-of-life care [36]. Previous studies showed that patients and the general public want to be involved in end-of life decision making rather than making their own decisions (shared decision making model). In a North American study, 71 % of 38 hospitalized elderly patients wanted to be involved in decision making (to decide when their quality of life is no longer acceptable, to ensure that every possible measure was taken to keep them alive), [25] another North American study on seriously ill patients found that 10 % preferred to leave all decisions to the doctor, 9 % that the doctor makes decision after considering their opinion, 32 % shared decision making, 24 % to make the final decision after considering the doctor opinion, and 16 % to make decision alone, [20] and 74 % of European general public [35] and about 50 % of Kenyan and Namibian general public wanted to be involved in decisions about their care [37].

### Value of Q-methodology in exploring end-of-life preferences

Development of an optimal instrument for studying end-of-life care continues to be a work in progress [53, 54]. Several items QODD items are missing from PADD (e.g., being mentally aware, peace with God) [14]. On the other hand, QODD does not include methods for importance weighting [41]. Our instrument has built on previous instruments and included statements related to Islamic Middle eastern culture and is applicable to forced-ranking and Q-methodology. With independent rating, respondents tend to attribute maximum importance to a large number of priorities, [14] which is avoided by forced-ranking, where items are evaluated relative to each other. Further, averaging analysis has a homogenization and depersonalizing effect, which is avoided by factor analysis and Q-methodology that has the advantage of quantifying minority groups.

We hypothesized that there are latent constellations of end-of-life choices that may be masked by averaging analysis. Indeed, we found that 29, 14, 14, 50, and 36 % of the extreme 14 priorities/dis-priorities for the five opinion types, respectively, were not among the extreme 20 priorities/dis-priorities identified by averaging analysis for the entire cohort. Using a smaller Q-set of 34 statements, a study on 37 South Korean university students’ perception of good death identified four types, a resolute acceptance type, a reasonable, natural life span type, a relational, sentimental type, and an altruistic, satisfied type [43].

### Study limitations

This study had a number of limitations. First, it was based on convenience sampling and performed at a single tertiary healthcare institution in a major metropolitan city. Further, because forced-ranking is mentally demanding, only educated and committed individuals were suitable for the study. Thus our results could be generalized only with caution even though the institution is a governmental referral center for the entire country.

Second, the study confronted rather healthy individuals with a hypothetical scenario. Responses to hypothetical scenarios may not accurately indicate what people would choose in real-life situations. However, such responses have enriched our understanding of end-of-life choices, [19, 32–37] as they likely reflect internalized range of norms and general beliefs in the society. Arguably, choices of terminally ill patients may be rather reactive, reflecting the desire of the immediate self rather than the planning, calculating self. Over a few months, half of the terminally ill patients who reported seriously considering euthanasia and physician-assisted suicide for unremitting pain changed their mind [21].

Third, Q-methodology is exploratory and not exhaustive in nature. Opinion types are defined as prototypical exemplars rather than as discrete categories; Q-methodology assumes neither discontinuous data nor clear cut-off points between categories. Thus the five opinion types extracted in this study should be seen as impressionistic conclusions. Further, it is likely that there are opinion types other than those identified in the study. Moreover, the prevalence of the identified opinion types among the larger population requires large sample surveys and standard analytic methods.

## Conclusions

Despite these limitations, our data support four main conclusions. 1) In young adult Saudi males transcendence was the extreme end-of-life priority, and dying in the hospital was the extreme dis-priority. 2) Quality of life was conceptualized differently with less emphasize on its physiological aspects. 3) Disclosure of terminal illness to family/close friends was preferred as long it is through the patient. 4) Q-methodology identified five types of constellations of end-of-life priorities and dis-priorities that may be related to respondents’ demographics and are partially masked by averaging analysis. The results emphasize the need to consider broader quality of life meanings as well as patients’ faith and religion to achieve the goal of quality death and dying. Patient-centered approaches to end-of-life care needs to take into account the identified priorities, dis-priorities, and typologies to sensitize health workers caring for Muslims/ Middle Eastern patients. This is the first study, to our knowledge to assess end-of-life priorities in such cultures; our findings need to be expanded and confirmed. The results also emphasize diversity in prioritizing end-of-life issues; one average opinion profile may not be useful as concerns can appear in varying combinations and hierarchy.

## Additional files

Additional file 1:
**Sorting Sheet & Instructions.** (DOC 50 kb) Additional file 2:
**Q-Set Domains.** (DOC 34 kb) Additional file 3:
**Factor Analysis.** (DOCX 27 kb)

## Abbreviations

PADD: Preference about dying and death
PRISMA: Positive diveRsities of European prIorities for reSearch and Measurement in end of life cAre
QODD: Quality of dying and death
