# Supplementary material for: Exploring end of life priorities in Saudi males: usefulness of Q-methodology
Source: BMC Palliat Care. 2015 Nov 26;14:66. doi: 10.1186/s12904-015-0064-5 (PMC4661936; doi:10.1186/s12904-015-0064-5)
Supplement: Additional file 1: — Sorting Sheet & Instructions. (DOC 50 kb) [file 12904_2015_64_MOESM1_ESM.doc]

**Instructions to Sorters**

Please read the following instructions carefully before you start your sort. It is very important that the sorting procedure be followed in all its details.

We ask you to indicate the degree of your agreement or disagreement with each of the statements in the deck of cards that was given to you. The 47 cards in the deck contain statements about issues related to end of life. We ask you to rank-order these statements according to your own point of view, using the attached Q-grid and Q-sorting sheet. The numbers on the cards (1 to 47) have been assigned randomly to the cards. They are used only to help you indicate your response.

Just to be clear, we are interested in your own point of view. Therefore, there is no right or wrong answer. We want to know what you think is most desired and what you think is most undesired, based on your own values and preferences. We are interested in your personal view, your view as an individual rather than your view as part of an organization, profession, or society. Your response will be treated as highly confidential and will be used only for the purpose of this study. It will not be linked to your medical record or used to inform your medical management.

Read through all the statements carefully to get a general impression of the range of issues at hand. You may notice that some of them are in agreement with your view, to a greater or lesser degree. Other statements may be in disagreement with your view, also to a greater or lesser degree. There are yet other statements that you may feel “neutral’ or ambivalent about. These possibly seem irrelevant to your own values and preferences.

Your task is to indicate the degree of agreement or disagreement with each statement. What is important to us is not *why* you consider a statement is agreeable or disagreeable but *how much* you agree or disagree with it. The statements should be sorted in an order of agreeability from your personal point of view; those most agreeable should be scored the highest, while those most disagreeable should be scored the lowest.

First, divide the statements into three piles, most agreeable, most disagreeable, and “others”. Then look through the most agreeable pile and pick out the three statements that are most agreeable to you. Place them in the three boxes under number “9” in the Q-sort grid. It does not matter which one goes on top or in the middle. Now, look through the same pile again and pick out the four statements that you consider most agreeable to you (excluding from consideration those statements you have already put under number “9”). Place them in the four boxes under number “8” in the Q-sort grid. Now of those statements that remain in the most agreeable pile, pick out six statements that you consider most agreeable to you (excluding from consideration those statements you have already put under numbers “9” and “8”). Place them in the six boxes under number “7” in the Q-sort grid.

Now work from the opposite end toward the middle. Of those statements in the most disagreeable pile, pick out the three statements that you consider most disagreeable to you. Place them in the three boxes under number “1” in the Q-sort grid. Now look through the same pile again and pick out the four statements that you consider most disagreeable to you (excluding from consideration those statements that you have already put under number “1”). Place them in the four boxes under number “2” in the Q-sort grid. Now of those statements that remain in the most disagreeable pile, choose the six statements that you consider most disagreeable to you. Place them in the six boxes under number “3” in the Q-sort grid.

Now you have to sort out the remaining 21 statements in the “others” pile. Pick up the 7 statements that you consider agreeable to you. Place them in the seven boxes under number “6” in the Q-sort grid. Pick up the 7 statements that you consider disagreeable to you. Place them in the seven boxes under number “4” in the Q-sort grid. Pick up the 7 statements that are left in the pile, place them under number “5” in the Q-sort grid.

You may have difficulty in placing the required number of statements into each of the 9 categories. For example, if 6 statements are required for a category, you may find that you have too many or too few. In either event, finish with the required number of statements, either by eliminating those that can most sensibly be moved out or by moving in those statements that are most relevant. You may feel that some of your placements are forced. Your task may be admittedly an awkward one, but try to work through it anyway. Before finalizing your Q-sort, make sure your preferences are reflected on the Q-sort grid in front of you. Feel free to rearrange any/all statements, so that when you are done, the positions of the statements relative to each other reflect how you feel, as closely as it can be. When you are done, please write down the number of each card in the boxes of the Q-sorting sheet that correspond to the boxes of the Q-sort grid that you have placed the cards in. The Q-sorting sheet has the same arrangement as the Q-sort grid. We will collect your Q-sorting sheet to analyze it. After writing the numbers of all the cards in the corresponding boxes of the Q-sorting sheet, please explain/give us your comments why the three statements you have placed under number “9” are the most agreeable to you and why the three statements you have placed under number “1” are the most disagreeable to you (please use the attached sheet). This will help us understand your preferences.

We would like to emphasize that the worth of this research is heavily dependent on how well and conscientiously participating people perform their tasks. Sorting the statements as described above is perhaps tedious. But when honestly done, the results would be very useful. On the other hand, analysis of statements that have been haphazardly positioned or positioned without due considerations would lead to wrong conclusions. Therefore, we would like to request that you return the material to the study coordinator without your response, if for any reason you feel that you cannot, or prefer not to, perform the task in a meaningful manner.

Thank you for your cooperation.

**Sorting Sheet**

Most disagreeable Most agreeable

| **1** | **2** | **3** | **4** | **5** | **6** | **7** | **8** | **9** |
| --- | --- | --- | --- | --- | --- | --- | --- | --- |
|  |  |  |  |  |  |  |  |  |
|  |  |  |  |  |  |  |  |  |
|  |  |  |  |  |  |  |  |  |
|  |  |  |  |  |  |  |  |  |
|  |  |  |  |  |  |  |  |  |
|  |  |  |  |  |  |  |  |  |
|  |  |  |  |  |  |  |  |  |

Please explain/comment why the three statements you have placed below number “9” are most agreeable to you.

Card #:____

_______________________________________________________________________

_______________________________________________________________________

Card #:____

_______________________________________________________________________

_______________________________________________________________________

Card #:____

_______________________________________________________________________

_______________________________________________________________________

Please explain/comment why the three statements you have placed below number “1” are most disagreeable to you.

Card #:____

_______________________________________________________________________

_______________________________________________________________________

Card #:____

_______________________________________________________________________

_______________________________________________________________________

Card #:____

_______________________________________________________________________

_____________________________________________________________________
