# Supplementary material for: Exploring end of life priorities in Saudi males: usefulness of Q-methodology
Source: BMC Palliat Care. 2015 Nov 26;14:66. doi: 10.1186/s12904-015-0064-5 (PMC4661936; doi:10.1186/s12904-015-0064-5)
Supplement: Additional file 2: — Q-Set Domains. (DOC 34 kb) [file 12904_2015_64_MOESM2_ESM.doc]

**Q-Set Domains**

The final Q-set consisted of 47 statements with 8 thematic domains: symptoms and personal control (7), treatment preferences (5), whole-person-concerns (8), moment of death (5), family/friends (6), achieving sense of completion/spirituality/religiosity (5), preparation for death (5), and relationship with healthcare professionals (6). The first three domains are most related to life quality vs quantity concerns, the fourth and fifth to connectedness, the sixth to transcendence, the seventh to coping, and the eighth to information-disclosure and decision-making. Each statement was randomly assigned a number from 1 to 47.

**Life quality vs. life quantity**

**Symptoms and personal control**

2- I want to die having no difficulty breathing

7- I want to die free of anxiety

8- I want to die free of pain

9- I want to die free of depression

30- I want to die being able to control my bladder

33- I want to die being able to bathe and feed myself

34- I want to die being able to control my bowels

**Treatment preferences**

1- I want to have no tubes inserted into my body

6-If I go into coma, I do not want to be placed in an intensive care unit

10- I want to receive all available treatments no matter what the chances of success are

11- I do not want to be kept on life support when there is little hope for a meaningful recovery

12- I want to live longer regardless of my medical condition

**Whole person concerns**

27- I want to die being able to communicate with others

31- I want to die at the peak of my life

35- I want to die maintaining my dignity

37- I want to die clean

41- I want to be referred to as a person not as a disease or a number

42- I want to die without having my body exposed

43- I want to die maintaining my sense of humor

44- I want to die well dressed

**Connectedness**

**Moment of death**

3- I want to die in the hospital

23- I do not want to die alone

26- I want to die at home

28- I want to have my family/ friends with me at my last moments

40- I want to have an Islamic clergy with me at my last moments

**Family/ friends**

13- I want my family/ friends rather than my doctor to inform me about my impending death

21- I want my doctor to discuss any concerns relating to my illness and care in the

presence of my family

22- I want my medical status to be kept confidential from my family/ friends

25- I want to avoid being an emotional burden to my family/ friends

29- I want to die knowing that my family/friends are prepared to accept my death

47- I want to avoid being a financial burden to my family/ friends

**Transcendence**

**Achieving sense of completion/spirituality/religiosity**

24- I want to resolve any conflict before I die

36- I want to die at peace with God

38- I want to die being able to say the statement of faith (*shahadah*).

39- I want my religious death rituals to be respected

45- I want to avoid being a financial burden to my society

**Coping**

**Preparation for death**

14- I want to discuss my fears about dying with my physician

15- I want to discuss my fears about dying with my family/friends

18- If I have a fatal illness, I don’t want to know

32- I want to die instantaneously

46- I want to have my financial affairs in order before I die

**Information-disclosure & decision-making**

**Relationships with health care professionals**

5- I want to receive medical care with compassion

4- I want to receive care from healthcare professionals whom I religiously trust

16- I want the doctor to inform me about my impending death before informing my family

17- I want to make my own medical decisions

19- I want to have my doctor available to answer my questions

20- I want to receive medical information regularly from medical staff
