# Supplementary material for: Exploring end of life priorities in Saudi males: usefulness of Q-methodology
Source: BMC Palliat Care. 2015 Nov 26;14:66. doi: 10.1186/s12904-015-0064-5 (PMC4661936; doi:10.1186/s12904-015-0064-5)
Supplement: Additional file 3: — Factor Analysis. (DOCX 27 kb) [file 12904_2015_64_MOESM3_ESM.docx]

**Table-S1:** Factor Loading Scores for 120 Respondents

| Sort | Factor-1 | Factor-2 | Factor-3 | Factor-4 | Factor-5 | Communality |
| --- | --- | --- | --- | --- | --- | --- |
| 1 | 33 | 29 | 39 | 36 | 5 | 49 |
| 2 | 20 | 67 | 28 | 11 | 7 | 59 |
| 3 | -11 | 56 | 5 | -1 | -10 | 33 |
| 4 | 51 | -2 | 20 | 34 | 11 | 42 |
| 5 | 10 | 8 | 68 | 26 | 17 | 57 |
| 6 | 10 | 66 | 14 | 19 | 10 | 51 |
| 7 | 2 | 8 | 5 | 56 | 11 | 34 |
| 8 | 7 | -2 | 52 | 27 | 20 | 38 |
| 9 | -1 | 52 | 39 | 56 | -2 | 73 |
| 10 | 22 | 26 | 42 | 36 | 2 | 43 |
| 11 | 34 | 23 | 50 | 17 | -4 | 45 |
| 12 | 40 | 5 | 30 | 42 | -11 | 46 |
| 13 | 45 | 26 | 5 | 0 | 28 | 35 |
| 14 | 20 | 13 | 5 | 41 | 56 | 54 |
| 15 | -2 | 36 | 69 | 19 | 5 | 66 |
| 16 | 16 | 55 | 14 | 36 | 1 | 48 |
| 17 | 14 | 60 | -4 | 26 | 13 | 48 |
| 18 | 40 | 2 | 45 | 1 | 2 | 37 |
| 19 | 60 | 19 | 19 | 5 | 5 | 45 |
| 20 | 2 | 17 | 47 | 61 | 8 | 65 |
| 21 | 29 | 30 | 64 | -2 | 10 | 60 |
| 22 | 37 | 20 | 47 | 17 | -12 | 45 |
| 23 | 38 | 26 | -2 | -11 | 31 | 32 |
| 24 | 32 | 8 | 23 | 46 | 17 | 40 |
| 25 | 56 | 26 | 16 | 2 | 20 | 46 |
| 26 | 37 | 56 | 16 | 17 | 9 | 51 |
| 27 | 34 | 45 | 30 | 34 | -5 | 53 |
| 28 | 4 | 14 | 11 | 35 | 28 | 24 |
| 29 | 19 | 19 | 56 | -4 | 19 | 42 |
| 30 | 5 | -8 | 39 | 2 | -40 | 33 |
| 31 | 2 | -7 | 75 | 11 | -11 | 59 |
| 32 | 30 | 19 | 46 | 19 | 28 | 46 |
| 33 | 5 | -4 | 30 | 17 | 52 | 40 |
| Sort | Factor-1 | Factor-2 | Factor-3 | Factor-4 | Factor-5 | Communality |
| 34 | -2 | 61 | 32 | -29 | 23 | 63 |
| 35 | 33 | -5 | 26 | 50 | 25 | 49 |
| 36 | 46 | 11 | 4 | 17 | 17 | 29 |
| 37 | 32 | 33 | 51 | 20 | 28 | 58 |
| 38 | 17 | 56 | 40 | -2 | 17 | 55 |
| 39 | 16 | 66 | 32 | 1 | 32 | 66 |
| 40 | 56 | 7 | 13 | 5 | 0 | 34 |
| 41 | 52 | 17 | 45 | 8 | -1 | 50 |
| 42 | 26 | 14 | 40 | 36 | 16 | 42 |
| 43 | 45 | 10 | 2 | 14 | 19 | 26 |
| 44 | 33 | 14 | 40 | 17 | 35 | 45 |
| 45 | 11 | 28 | 56 | 20 | 5 | 45 |
| 46 | 8 | 25 | 51 | 32 | 16 | 45 |
| 47 | 17 | 40 | 44 | 16 | 13 | 43 |
| 48 | 34 | 2 | 40 | -16 | 40 | 46 |
| 49 | 14 | 40 | 14 | 52 | 5 | 48 |
| 50 | -8 | 26 | 23 | 11 | 5 | 15 |
| 51 | 4 | 46 | 29 | 58 | 16 | 68 |
| 52 | 26 | 52 | 34 | 5 | 14 | 48 |
| 53 | 39 | 4 | 35 | 11 | 17 | 32 |
| 54 | -23 | 44 | 42 | 27 | 22 | 55 |
| 55 | 2 | 44 | 23 | 4 | 20 | 29 |
| 56 | 4 | 2 | 2 | 27 | 69 | 56 |
| 57 | 5 | 41 | 46 | 41 | -13 | 58 |
| 58 | 25 | -2 | -8 | 19 | 33 | 21 |
| 59 | 32 | 55 | 29 | 14 | 8 | 52 |
| 60 | 38 | 30 | 7 | -13 | 25 | 32 |
| 61 | 50 | 4 | 26 | 17 | 17 | 37 |
| 62 | -2 | 48 | 36 | 2 | 13 | 39 |
| 63 | 2 | 23 | 54 | 14 | 22 | 42 |
| 64 | 2 | 33 | 47 | 28 | 11 | 43 |
| 65 | 32 | 1 | 66 | 14 | 2 | 56 |
| 66 | 45 | 14 | -10 | 5 | 51 | 49 |
| 67 | 13 | 45 | 47 | 42 | -5 | 63 |
| Sort | Factor-1 | Factor-2 | Factor-3 | Factor-4 | Factor-5 | Communality |
| 68 | 14 | 25 | 36 | 22 | 22 | 31 |
| 69 | 10 | 30 | 26 | 30 | 28 | 35 |
| 70 | 16 | 8 | 46 | 25 | 11 | 32 |
| 71 | 13 | 45 | 50 | 34 | -25 | 64 |
| 72 | 4 | 64 | 11 | 22 | 1 | 47 |
| 73 | 48 | 30 | 56 | 27 | -5 | 73 |
| 74 | 8 | 34 | 28 | 5 | 40 | 37 |
| 75 | 8 | 35 | 26 | 32 | 33 | 41 |
| 76 | 28 | 7 | 57 | 27 | 11 | 50 |
| 77 | 19 | 25 | 47 | -10 | 19 | 37 |
| 78 | 29 | 20 | 56 | 5 | 23 | 49 |
| 79 | 0 | 40 | -8 | 14 | 14 | 20 |
| 80 | 30 | 44 | 40 | -5 | 22 | 50 |
| 81 | -8 | 11 | 25 | 17 | -25 | 17 |
| 82 | 10 | 23 | 60 | 30 | 5 | 53 |
| 83 | 22 | 5 | 36 | 11 | 40 | 36 |
| 84 | 23 | 20 | 13 | 0 | 55 | 42 |
| 85 | 44 | 0 | 56 | 14 | 11 | 54 |
| 86 | 20 | 25 | 14 | 42 | 34 | 43 |
| 87 | 26 | 50 | 40 | 8 | -4 | 49 |
| 88 | 7 | 11 | 41 | 28 | 5 | 27 |
| 89 | 8 | 41 | -16 | 28 | 8 | 30 |
| 90 | 47 | 13 | 40 | 8 | -8 | 43 |
| 91 | 14 | 17 | 56 | 11 | 29 | 47 |
| 92 | 0 | 32 | 14 | 0 | 57 | 45 |
| 93 | 14 | 11 | 26 | 11 | 40 | 27 |
| 94 | 28 | 20 | 65 | -1 | 14 | 56 |
| 95 | 16 | 63 | 39 | -1 | -23 | 62 |
| 96 | 32 | 27 | 50 | 40 | 10 | 59 |
| 97 | 13 | 45 | 46 | 4 | 20 | 47 |
| 98 | 11 | 40 | 46 | 20 | 2 | 43 |
| 99 | 59 | 27 | 14 | 28 | 1 | 53 |
| 100 | 7 | 48 | 56 | -2 | 26 | 63 |
| 101 | 26 | 0 | 5 | 38 | -8 | 22 |
| Sort | Factor-1 | Factor-2 | Factor-3 | Factor-4 | Factor-5 | Communality |
| 102 | 7 | 13 | 32 | 33 | 17 | 26 |
| 103 | 29 | 42 | 13 | 17 | 33 | 42 |
| 104 | 28 | 22 | 8 | 44 | 7 | 33 |
| 105 | 19 | 34 | 44 | 11 | -28 | 43 |
| 106 | 51 | 4 | 59 | -7 | 5 | 63 |
| 107 | 17 | 34 | 20 | 38 | 34 | 45 |
| 108 | -4 | 30 | 40 | 11 | 8 | 27 |
| 109 | 30 | 20 | 25 | 47 | -1 | 43 |
| 110 | 23 | -1 | 42 | 28 | 7 | 32 |
| 111 | 28 | 35 | 13 | 19 | 42 | 45 |
| 112 | 11 | 17 | 23 | 23 | 19 | 19 |
| 113 | -14 | 17 | 16 | 32 | 40 | 33 |
| 114 | 41 | 32 | 34 | 2 | 17 | 42 |
| 115 | 19 | 30 | 29 | 14 | 47 | 47 |
| 116 | 8 | 17 | 28 | 36 | 34 | 38 |
| 117 | 44 | 0 | 5 | 39 | 19 | 38 |
| 118 | -11 | -2 | 17 | 65 | 29 | 56 |
| 119 | -1 | 29 | 55 | 34 | 4 | 51 |
| 120 | 25 | 40 | 39 | 7 | 39 | 53 |

**Table S2:** Factor Scores

|  | Factor-1 | Factor-2 | Factor-3 | Factor-4 | Factor-5 | Total |
| --- | --- | --- | --- | --- | --- | --- |
| Eigenvalue | 8.9 | 12.4 | 17.2 | 8.6 | 6.9 | 54.0 |
| % Variance | 7 | 10 | 14 | 7 | 6 | 44 |

**Table S3:** Factor Correlations

|  | Factor-1 | Factor-2 | Factor-3 | Factor-4 | Factor-5 |
| --- | --- | --- | --- | --- | --- |
| Factor-1 | - | 59 | 54 | 42 | 40 |
| Factor-2 | 59 | - | 52 | 38 | 42 |
| Factor-3 | 54 | 52 | - | 46 | 46 |
| Factor-4 | 42 | 38 | 46 | - | 44 |
| Factor-5 | 40 | 42 | 46 | 44 | - |

**Table S4:** Consensus Statements

| Statement | Factor-1 | Factor-2 | Factor-3 | Factor-4 | Factor-5 |
| --- | --- | --- | --- | --- | --- |
| I want my medical status to be kept confidential from my family/friends. | 2 | 2 | 2 | 2 | 2 |
| I want to die at peace with God. | 9 | 9 | 9 | 9 | 9 |
| I want to die being able to say the statement of faith (shahadah). | 9 | 9 | 9 | 9 | 9 |

**Table S5:** Differentiating Statements

| Statement | Factor-1 | Factor-2 | Factor-3 | Factor-4 | Factor-5 |
| --- | --- | --- | --- | --- | --- |
| I want to have an Islamic clergy with me at my last moments. | 1 | 7 | 5 | 6 | 6 |
| I want to die being able to bathe and feed myself. | 3 | 5 | 9 | 4 | 3 |
| I want to die being able to control my bladder. | 5 | 6 | 7 | 6 | 1 |
